# Effect of Periodic Water Clusters on AISI 304 Welded Surfaces

**DOI:** 10.3390/ma14010210

**Published:** 2021-01-04

**Authors:** Madhulika Srivastava, Akash Nag, Lucie Krejčí, Jana Petrů, Somnath Chattopadhyaya, Sergej Hloch

**Affiliations:** 1 Department of Mechanical Engineering, Amrita School of Engineering, Amrita Vishwa Vidyapeetham, Chennai 601103, India; s_madhulika@ch.amrita.edu; 2 Department of Mechanical Engineering, Indian Institute of Technology (Indian School of Mines), Dhanbad 826004, India; akashnag1992@gmail.com (A.N.); somnathchattopadhyaya@iitism.ac.in (S.C.); 3 Department of Mechanical Engineering, Vysoká Škola Báňská, Technical University, 708 00 Ostrava, Czech Republic; lucie.krejci@vsb.cz (L.K.); jana.petru@vsb.cz (J.P.); 4 Faculty of Manufacturing Technologies, Technical University of Kosice, Prešov, Bayerova 1, 080 01 Presov, Slovakia; 5 Institute of Geonics of the Czech Academy of Sciences, Studentska 1768, 708 00 Ostrava-Poruba, Czech Republic

**Keywords:** pulsating water jet, welded joints, stainless steel, residual stress, microhardness, surface roughness

## Abstract

This study compared the effect of the interaction time of periodic water clusters on the surface integrity of AISI 304 tungsten inert gas (TIG) welded joints at different excitation frequencies, as the effect of the technological parameters of pulsating water jet (PWJ) on the mechanical properties of TIG welded joints are under-researched. The TIG welded joints were subjected to different frequencies (20 and 40 kHz) and traverse speeds (1–4 mm/s) at a water pressure of 40 MPa and a standoff distance of 70 mm. The effect of the interaction of the pulsating jet on the material and the enhancement in its mechanical properties were compared through residual stress measurements, surface roughness, and sub-surface microhardness. A maximum enhancement in the residual stress values of up to 480 MPa was observed in the heat-affected zone, along with a maximum roughness of 6.03 µm and a maximum hardness of 551 HV using a frequency of 40 kHz. The improvement in the surface characteristics of the welded joints shows the potential of utilizing pulsed water jet technology with an appropriate selection of process parameters in the treatment of welded structures.

## 1. Introduction

The phenomenon of hydrodynamic erosion has gained increasing attention due to its severe effects on the hydraulic mechanical components such as impellers, valves, fittings, pumps, and hydrofoil surfaces [1,2]. The erosion initiated by impact loading, cyclic loading in the form of a hammer effect, and lateral jetting causes elastic-plastic deformation during the interaction of the jet with the material [3]. Fundamentally, the impact of a water jet or droplet on a solid surface generates stresses that are categorized into two stages. The initial stage is the water hammer effect, which causes compressive stress for a short duration, followed by the shearing action of the material during lateral jetting [4]. The compressive and shear stresses are distributed within the surface and sub-surface layers in the form of shock waves, which are responsible for enhancing the strength and surface integrity of the material. The conclusions inferred from previous experiments suggest that the erosion effect is the consequence of fatigue failure caused by the impact of a jet [5]. The energy of a jet imparted on a solid surface is utilized for treatment applications by exploring the early stages of erosion (incubation). However, at the advanced stages of erosion, disintegration of the material occurs.

In view of the above, the erosion phenomenon (early and advanced stages of erosion) has been investigated using a pulsating (PWJ) or continuous (CWJ) water jet on various ductile materials. The water droplet erosion (WDE) mechanism has been investigated on Ti–Al alloys (fully lamellar) [6], during which the test was interrupted at three stages of erosion (i.e., the incubation, maximum erosion, and steady-state stages); the microstructural analysis revealed that the erosion was initiated through inhomogeneous and localized material flow, followed by crack generation on the surface. Moreover, the advanced stages of erosion were directed by the periodic roughening and formation of deep craters. The WDE (early and advanced stages) has also been studied on Ti–6Al–4V surfaces [7], in which the influence of the impact speed on erosion behavior in terms of mass loss was explored. It was observed that at impact speed (350 m/s), the maximum erosion rate increases as the initiation time becomes faster. The microstructural analysis revealed that the early erosion was limited to microcracks and isolated pits; however, substantial material removal (sub-surface cracking, upheaving) was observed in the advanced stage of erosion. Additionally, the effect of a water jet has also been explored for surface treatment application on various ductile materials, such as aluminum alloys [8], in which the effect of various process parameters (e.g., pressure, number of passes, standoff distance, and feed rate) on the surface roughness and microhardness were investigated. The experimental analysis revealed that an increase in the number of passes (1 to 3), pressure (50–150 MPa), and standoff distance (20–60 mm) results in higher roughness and hardness values; however, a reverse effect was observed upon increasing the feed rate. Another study [9] investigated the effect of the number of passes, the pressure, and the feed rate on the surface integrity of stainless steel. An enhancement in erosion and roughness was revealed upon increasing the number of passes and the pressure; however, the feed rate showed a reverse effect on the surface erosion and roughness.

Under the action of varying loads (impact and lateral jetting) on the material, modulated water jets are more suitable for material processing in comparison to CWJs [3]. Modulated jets can be generated using different methods, such as self-resonating nozzles, interrupting continuous jets by a rotating disc with an orifice, or using vibrating mechanical devices (e.g., needle or ultrasonic cylinder) inside the pressure chamber. The impact of modulated jets generated using the above methods induces cyclic loading on the material surface [10]. However, the related drawbacks, such as the short life of the moving components and design complexities, are overcome by using ultrasonic generator methods of pulse generation. This technology utilizes a repeated hammer effect produced by the periodic motion of the sonotrode and is advantageous in terms of the lifetime and reliability of the system [10].

The interaction of a PWJ in terms of parametric influence (i.e., pressure, traverse speed, and standoff distance) in the early and advanced stages of erosion [11,12,13,14] and the flow field characteristics [15] has been explored previously. Moreover, to eliminate the tensile stresses induced in AISI 304 tungsten inert gas (TIG) welded joints [16], the weld surface has been exposed to a PWJ under variable parametric levels [17]. However, the effect of a variable interaction time and an excitation frequency at the same volumetric flow rate on welded joints has still not been effectively determined. Therefore, in the present study, the effect of different interaction times obtained by varying the nozzle traverse speed (1–4 mm/s) with different excitation frequencies (20, 40 kHz) on the surface integrity of welded AISI 304 was studied. The surface integrity was studied using residual stress, microhardness, and surface roughness measurements of the sample after PWJ treatment.

The outline of the study is divided into following sections: The introduction is followed by Section 2 (Materials and Methods) which illustrates the sample preparation and the experimental conditions along with the description of the setup used for the experiments and subsequent analysis Section 3 includes the results and the detailed discussion of the results obtained and Section 4 summarizes the primary conclusions of the investigation.

## 2. Materials and Methods

**Figure 1:**
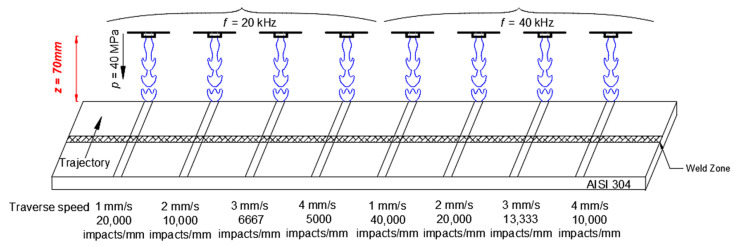
Experimental setup.

As done previously, double-butt TIG welded joints (Figure 1) of AISI 304 were fabricated at a current of 97 A with a voltage of 12.1 V for inner butts and 11.8 V for outer butts [17]. The chemical composition and mechanical properties of AISI 304 are mentioned in Table 1 and Table 2 [17]. The joints were then treated using an ultrasonically generated PWJ machine comprising a Hammelmann HDP 253 plunger pump (Hammelmann GmBH, Oelde, Germany) integrated with an ABB robot IRB 6640-180/2.55 (ABB s.r.o, Ostrava, Moravská Ostrava, Czech Republic) for handling the PWJ head and an ECOSON WJ-UG_630-40 (Ecoson s.r.o, Nové Mesto nad Váhom, Slovakia) sonotrode for the initiation of the PWJ at frequencies of 20 and 40 kHz. The system of the PWJ consisted of an acoustic generator with a cylindrical waveguide located inside the acoustic chamber. In this system, the electric signals are transmitted to the liquid inside the chamber in the form of mechanical vibrations from the sonotrode (via the aid of a piezoelectric transducer). These periodic oscillations of the sonotrode cause pressure fluctuations, which are amplified in the mechanical amplifier and guided through the nozzle exit. At the nozzle exit, the pressure fluctuations are converted to velocity fluctuations, causing the breakage of a continuous jet stream into discrete clusters. The repeated impact of these clusters on the material surface induces cyclic stress, which surpasses the ultimate strength of the material and results in elastic–plastic deformation. These deformations are responsible for improving the surface characteristics of the material [4].

A Stonage nozzle was selected for the treatment under the parameters stated in Table 3. Experiments at a frequency of *f* = 40 kHz were conducted under the experimental conditions mentioned in Table 1, after *f* = 20 kHz, which had already been reported earlier [17] (Figure 1). AISI 304 weld zone were exposed to PWJ starting with a traverse speed *v* = 1 mm/s increasing up to 4 mm/s. This variation in the traverse also varies the number of impingements from 20,000 to 4000 impacts per mm. The same sequence was followed with higher excitation frequency *f* = 40 kHz, as shown in Figure 1. Standoff distance and supply pressure for all experimental runs were kept constant at *z* = 70 MPa and *p* = 40 MPa.

**Figure 2:**
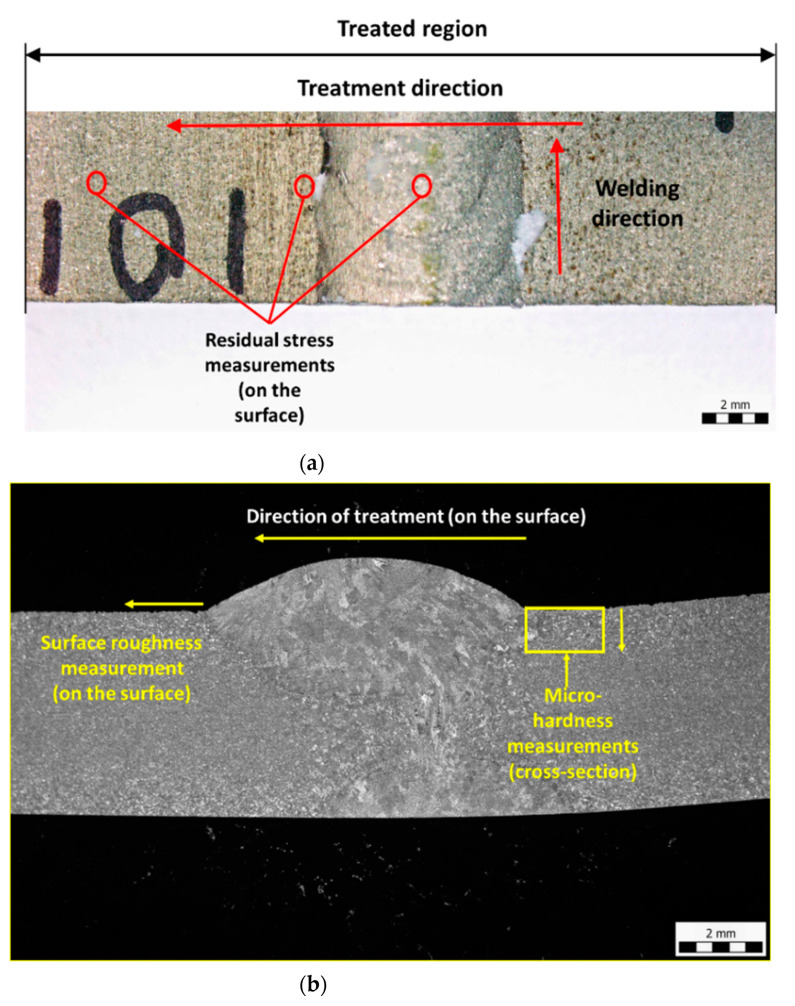
Optical Microscopy (Macro structure) of (**a**) the surface of the sample and (**b**) a cross-section of the sample showing the direction and regions selected for the measurements.

Figure 2a shows the treatment region and welding direction. The treatment was conducted on the surface along the width (50 mm) of the sample with a consecutive spacing of 5 mm between the traces to prevent the overlapping of the treated region. For observing the effect of interaction time of the jet with the AISI welded joint surface, a traverse speed of *v* = 1, 2, 3, and 4 mm/s was used.

The surface residual stress (Figure 2a) was measured using X-Ray Diffraction technique (XRD, PROTO Manufacturing Inc., Taylor, MI, USA). The conventional sin 2*Ψ* method, where the d-spacing is given as a function of sin 2*Ψ* based on an elliptical regression plot, was used to calculate the residual stress (XRD Win 2.0 software). The Mn-Kα X-ray tube of a 2 mm diameter was used for the measurements. The diffraction shift {311} was recorded at each point at rotation angles of 0°, 45°, and 90° with tilt phi angles at *Ψ* = ±30°. The Bragg’s angle was measured as 152.80°. The measurements were conducted on the surface in the three different welded zones (heat affected zone (HAZ), weld, and base) marked in Figure 2a.

The surface roughness of the treated region (Figure 2b) was quantified using a contact surface roughness tester (Make: Mitutoyo, Kawasaki, Kanagawa, Japan). A stylus of 5 µm diameter was traversed with a cutoff length of *l_c_* = 0.8 mm. The measured values (repeated five times) are plotted with mean and standard deviation. The roughness was measured only in the HAZ region.

The microhardness measurements were obtained from the sub-surface region of the polished samples (mirror-finished), starting from distance of 20 µm from the surface until a 1000 µm depth using the Vickers Hardness Tester at a load of 10 gf for a 10 s indentation time. The direction and the location of the measurements are shown in Figure 2b.

The plastic deformation phenomenon in the treated samples was observed using optical microscopy (Figure 2b). Before conducting the optical microscopy, a cross-section of the samples was polished and then etched using Adler reagent.

## 3. Results

### 3.1. Surface Residual Stress Measurements

**Figure 3:**
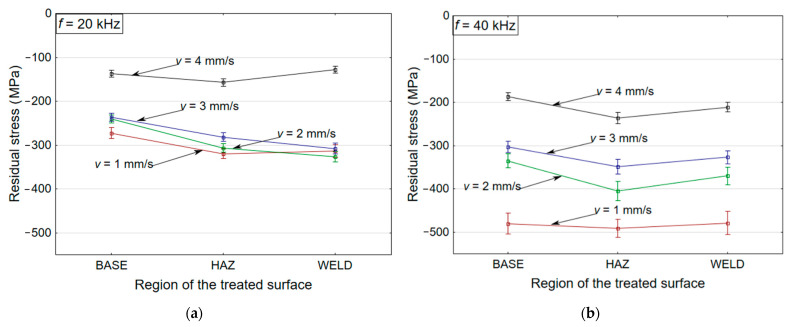
Variation in the maximum principal residual stress with varying traverse speeds in the different zones of the welded joints at frequencies of (**a**) *f* = 20 kHz [17] and (**b**) *f* = 40 kHz.

The residual stress data calculated using XRD measurements are plotted in Figure 3a,b at frequencies of *f* = 20 and 40 kHz. The deviation of the principal residual stress (maximum) under different treatment conditions at two frequency levels (*f* = 20 and 40 kHz) is shown, along with changes in the traverse speed (*v* = 1–4 mm/s) in the different zones of the welded joints. A noteworthy change in the residual stress conditions of the material was recorded between the two frequency levels.

At increased frequency levels of *f* = 40 kHz, a higher compressive residual stress of −480 MPa was recorded at a lower traverse speed (*v* = 1 mm/s) in comparison to the lower frequency level of *f* = 20 kHz (–272 MPa). Differences in the residual stress levels were recorded due to the successive increase in the number of impacts, from 20,000 impacts per mm (at *f* = 20 kHz) to 40,000 impacts per mm (at *f* = 40 kHz). This causes a successive increment in the propagation of the compressive and shear stresses (in the form of longitudinal and transverse waves) during the impact and release phases of the shock envelope [18]. The propagation of these different directional waves along the sub-surface of the impacted zone allows dislocations to move and increases in dislocation density [18], which enhances plastic deformation and thus results in an increased magnitude of compressive residual stresses (herein, from −319 MPa at *f* = 20 kHz to −480 MPa at *f* = 40 kHz). Primarily, dislocation motion occurs due to crystalline defects in the form of slip bands and twinning, which are responsible for the induction of residual stresses (compressive) during the treatment process [19]. The distribution of the residual stress (compressive) is determined by the parametric conditions.

It is also evident from Figure 3 that with the increase in traverse speed (*v* = 1–4 mm/s), the induction of compressive residual stresses decreased (from −319 MPa to −157 MPa at *f* = 20 kHz and from −479 MPa to −236 MPa at *f* = 40 kHz in HAZ) in all the three zones (i.e., weld, HAZ, and base). The reduction in the residual stresses is dependent on the coverage time of the interacting jet, which limits the propagation of compressive and shear stress along the sub-surface layers.

### 3.2. Surface Roughness and Microhardness Measurements

**Figure 4:**
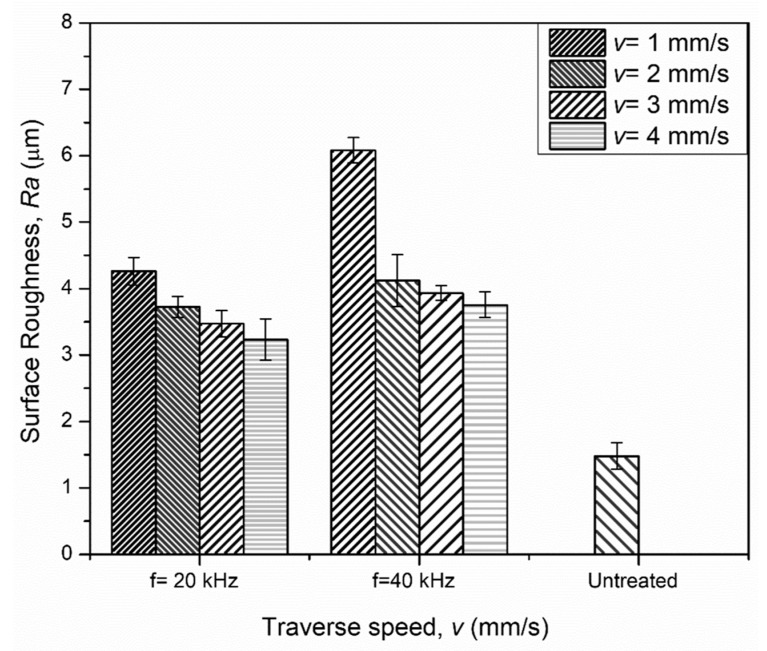
Variation in surface roughness at frequencies of *f* = 20 kHz [17] and *f* = 40 kHz at different traverse speeds.

Surface roughness significantly affects the performance of engineering components. It depends on the number of surface impingements that cause localized plastic deformation. The graph shown in Figure 4 depicts the effect of frequency variation (*f* = 20 and 40 kHz) with an average surface roughness (*Ra*), along with changes in the traverse speed. It was noted that the untreated roughness (*Ra*) of 1.48 µm increased to a maximum of 6.08 µm at the higher frequency of 40 kHz and at a traverse speed of *v* = 1 mm/s. The increased roughness values are attributed to the increase in the number of impacts from 20,000 to 40,000 impacts per mm, which allowed severe plastic deformation at a higher frequency level. Moreover, at the lower traverse speed of *v* = 1 mm/s, higher roughness values were recorded (*Ra* = 4.26 µm at *f* = 20 kHz and *Ra* = 6.083 µm at *f* = 40 kHz) in comparison to the higher traverse speed of *v* = 4 mm/s. This is due to the contact time of the interacting jet, which determines the duration of shock wave propagation beneath the treated region.

The cross-sectional analysis of the treated region revealed the effect of the treatment process on the strength of the material as a result of non-uniform plastic deformation [20]. Generally, plastic deformation in metals involves the motion of dislocations. These dislocations are more prominent on the surface areas adjacent to the impacted surface. During plastic deformation, these dislocations in the impacted region are obstructed by the motion of adjacent dislocations, thus causing hardening or strengthening of the material [9].

**Figure 5:**
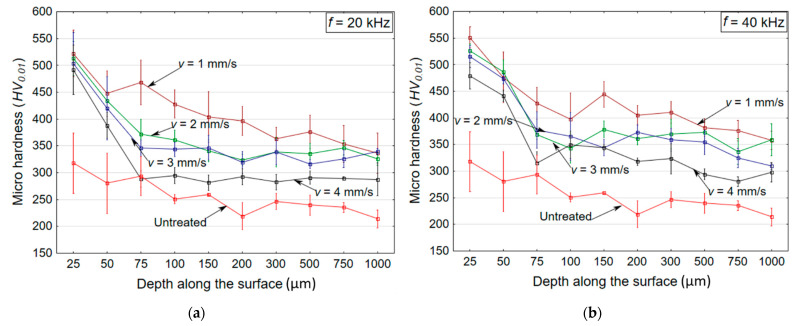
Variation in the microhardness along the depth at (**a**) *f* = 20 kHz and (**b**) *f* = 40 kHz.

The measurements were carried out in each sample along the depth (from 20 to 1000 µm). The hardness values are plotted in the graph in Figure 5 with depth variation for the untreated and treated samples (in HAZ) at *p* = 40 MPa and *f* = 20 or 40 kHz in Figure 5a,b, respectively. A substantial increase in hardness was observed near the treated region in all samples, in contrast to the results of the untreated sample. The untreated sample showed a microhardness value of 318 HV, which was enhanced up to 551 HV after the treatment. It is evident from Figure 4 that the maximum increase in the hardness of the treated surface was obtained up to a depth of ~100 µm at both frequency levels (*f* = 20 and 40 kHz). At the higher frequency level of *f* = 40 kHz, higher hardness values (up to 551 HV) were measured owing to the enhanced number of impacts (from 20,000 to 40,000 impacts per mm at *v* = 1 mm/s). The increase in the number of impacts (40,000 impacts per mm) allowed for the enhanced propagation of compressive stress and strengthening of the layer beneath the impacted surface through the motion of dislocations. These results also agree with the residual stress values measured on the surface of the HAZ. It is also noted that, at the lower traverse speed of *v* = 1 mm/s, higher hardness values (551 HV) were recorded, due to the difference in the duration for which the interacting jet was incident on the sample surface, which induced higher values of residual stress and microhardness.

### 3.3. Optical Microscopy

**Figure 6:**
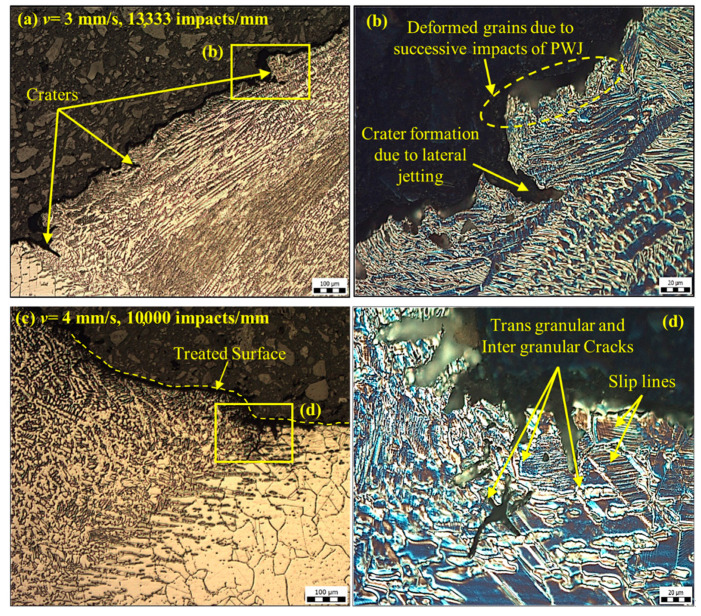
Optical microscopy of a cross-section of the samples treated by pulsating water jet (PWJ) at *f* = 40 kHz (**a**) at *v* = 3 mm/s (100 µm), with (**b**) a magnified view at 20 µm, and (**c**) at *v* = 4 mm/s (100 µm), with a (**d**) magnified view at 20 µm.

**Figure 7:**
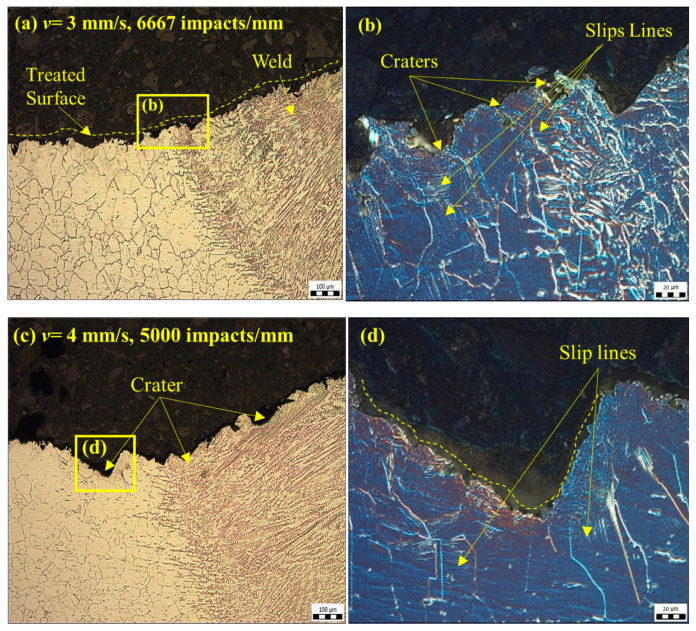
Optical microscopy of a cross-section of the samples treated by PWJ at *f* = 20 kHz (**a**) at *v* = 3 mm/s (100 µm), with (**b**) a magnified view at 20 µm, and (**c**) at *v* = 4 mm/s (100 µm), with a (**d**) magnified view at 20 µm. [17].

The polished samples after etching were subjected to an optical microscopy examination to elucidate the effect of plastic deformation along the cross-section of the samples. Figure 6 and Figure 7 illustrate the state of plastic deformation caused under the variation in the number of impacts (or frequency change from *f* = 20 to 40 kHz). At *f* = 40 kHz, deformation was observed in the form of craters and deformed grains. These features are the outcome of the repeated impact of the clusters of the PWJ (maximum 6667 (20 kHz)—13,334 impacts per mm at *v* = 3 mm/s). The impact of the jet on the materials caused stress distribution and shock wave propagation. On striking the surface asperities, higher impact pressures were generated and the energy accumulated in the weaker sections (grain boundaries and pits) of the material. The repeated impact prompted failure due to the interaction between the impact and the reflected waves, and resulted in the formation of cracks, craters, and deformed grains. The lateral jetting after the initial impact phase was responsible for the formation of craters (Figure 6b). Moreover, throughout the propagation of the stress waves within the material, dislocations moved and were occasionally hindered due to the presence of other dislocations in the lattice. The hindrance between the dislocations was responsible for the strengthening of the material [21]. Dislocations in the form of slip lines are evident in Figure 6d.

At *f* = 20 kHz, the presence of impact craters along with sub-surface deformities and slip lines with no splintering of grains is clearly evident in Figure 7. On comparing the intensity of the deformation, due to the increase in the number of impacts from ~6667 to 13,334 impacts per mm (at *v* = 3 mm/s) at the higher frequency *f* = 40 kHz, severe sub-surface deformation features such as intergranular and transgranular crack formation were observed, along with the presence of slip lines. These features were observed due to the enhanced compressive stress and shear stress propagation along the highly stressed regions when a larger number of impacts were incident on the surface.

**Figure 8:**
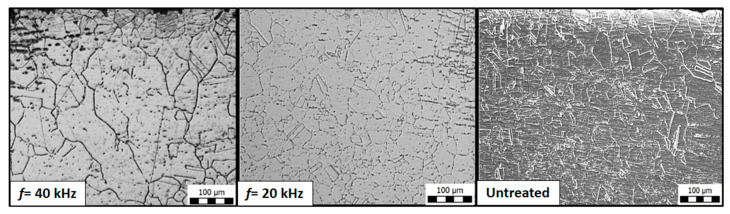
Optical microscopy of a cross-section of the samples in the HAZ at 100 µm.

**Figure 9:**
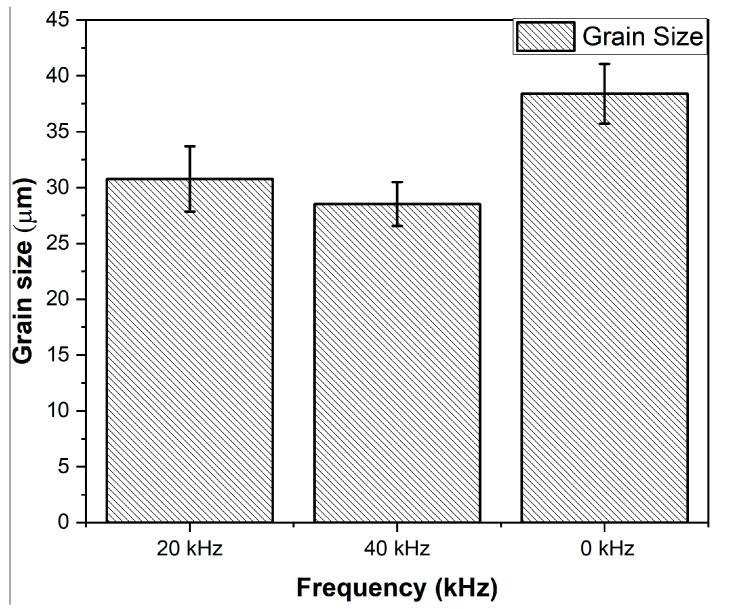
Grain size as a function of frequency.

Figure 8 shows the grains observed through optical microscopy along the cross-section of a sample in the HAZ. The grain size was measured in the treatment direction and the grain size was plotted as a function of the frequency (Figure 9). Samples treated at different frequencies (0, 20, and 40 kHz) with a constant traverse speed of *v* = 4 mm/s were used for this evaluation.

It is evident from Figure 9 that upon increasing the frequency from 0 to 40 kHz, finer grains were observed. The original grain (untreated or 0 kHz) of 38.40 µm was refined to 28.53 µm (minimum) at a frequency of 40 kHz. This effect is attributed to the phenomenon of grain boundary reforming [21]. The repeated hammering effect by the clusters of the jet caused plastic deformation in the sub-surface regions due to the propagation of the directional waves within the material. This propagation initiated the motion of dislocations and allowed them to rearrange and form new sub-boundaries. The successive impacts of the clusters increased the density of the dislocations and promoted the reformation of the grain boundaries. At the increased frequency level of *f* = 40 kHz, as the number of impacts increased (from 5000 impacts per mm at *f* = 20 kHz to 10,000 impacts per mm at *f* = 40 kHz for *v* = 4 mm/s), a greater hammering effect occurred on the surface, leading to grain refinement, resulting in smaller grain sizes (28.53 µm) than those obtained at *f* = 20 kHz (30.77 µm).

## 4. Conclusions

This study compared the effect of the excitation frequency on the surface integrity of AISI 304 TIG welded joints at varying nozzle traverse speeds. The effects were compared through residual stress, surface roughness, microhardness measurements, and optical analysis of the weldment. The obtained results can be summarized as follows:

The residual stress measurements showed that the initial condition of −122 MPa in HAZ improved to a maximum of −480 MPa at *f* = 40 kHz and of −319 MPa at *f* = 20 kHz at a lower traverse speed (*v* = 1 mm/s). It was also observed that the residual stress (compressive) distribution could be determined through the process parameters and material characteristics.

The surface roughness (*Ra*) value recorded in the untreated sample was 1.48 µm, which was increased to a maximum of 6.08 µm at the higher frequency of *f* = 40 kHz at *v* = 1 mm/s. This was attributed to the coverage time of the interacting jet.

The initial microhardness of the untreated welded joints was 318 HV, which increased to a maximum of 551 HV at *f* = 40 kHz. Moreover, the maximum increase in microhardness was observed at a lower traverse speed (*v* = 1 mm/s). A significant increase in hardening up to a depth of 100 µm was obtained at both frequency levels (*f* = 20 and 40 kHz).

The optical microscopy showed the presence of surface features such as slip bands and twin boundaries predominantly in the sub-surface region in comparison to the untreated samples. This indicates the intensity of the deformation under various experimental conditions. Additionally, the initial grain size of 38.40 µm was refined by 19.86% (30.77 µm) at *f* = 20 kHz and by 25.7% (28.53 µm) at *f* = 40 kHz.

The present study revealed the effect of the technological parameters, excitation frequency, and traverse speed of PWJ on TIG welded stainless steel surface. The enhancement in the mechanical properties shows the probability of utilizing the technology for the surface treatment application as it will be helpful in determining the appropriate parameters. However, for the surface treatment application the effect of variation of parameters should be explored in detail to ensure the surface integrity of the treated surface.

## Figures and Tables

**Table 1:** Composition of AISI 304 [17].

Element	C (wt.%)	Mn (wt.%)	S (wt.%)	Si (wt.%)	P (wt.%)	Ni (wt.%)	Cr (wt.%)
SS (AISI 304)	0.08	2.00	0.03	1.0	0.04	8–10.5	18–20

**Table 2:** Mechanical properties of AISI 304 [17].

STN	Grade	Tensile Strength (MPa)	Yield Strength (MPa)	Elongation (%)	Brinell Hardness	Structure
17.240	304	500	210	45	88	Austenitic

**Table 3:** Experimental conditions.

S. No.	*f* (kHz)	*p* (MPa)	*d* (mm)	*z* (mm)	*v* (mm/s)	No. of Impacts/mm	Impact Speed (m/s)	Impact Pressure (MPa)	Time Period of Impact Pulse (µs)
1	20 [17]	40	1.9	70	1	20,000	254.81	938.47	0.0466
2					2	10,000			
3					3	6667			
4					4	5000			
5	40				1	40,000			
6					2	20,000			
7					3	13,333			
8					4	10,000			

## Data Availability

The data presented in this study are available on request from the corresponding author.
